# Patients in complete remission after R-CHOP(-like) therapy for diffuse large B-cell lymphoma have limited excess use of health care services in Denmark

**DOI:** 10.1038/s41408-022-00614-8

**Published:** 2022-01-27

**Authors:** Lasse Hjort Jakobsen, Andreas Kiesbye Øvlisen, Marianne Tang Severinsen, Joachim Bæch, Kristian Hay Kragholm, Ingrid Glimelius, Anne Ortved Gang, Judit Mészáros Jørgensen, Henrik Frederiksen, Christian Bjørn Poulsen, Michael Roost Clausen, Per Trøllund Pedersen, Robert Schou Pedersen, Christian Torp-Pedersen, Sandra Eloranta, Tarec Christoffer El-Galaly

**Affiliations:** 1 Department of Hematology, Clinical Cancer Research Center, Aalborg University Hospital, Aalborg, Denmark; 2 Department of Clinical Medicine, Aalborg University, Alborg, Denmark; 3 Unit of Clinical Biostatistics and Epidemiology, Aalborg University Hospital, Aalborg, Denmark; 4 Department of Cardiology, Aalborg University Hospital, Aalborg, Denmark; 5 Clinical Epidemiology Division, Department of Medicine Solna, Karolinska Institutet, Stockholm, Sweden; 6 Department of Immunology, Genetics and Pathology, Unit of Oncology, Uppsala University, Uppsala, Sweden; 7 Department of Hematology, Rigshospitalet, Copenhagen, Denmark; 8 Department of Hematology, Aarhus University Hospital, Aarhus, Denmark; 9 Department of Hematology, Odense University Hospital, Odense, Denmark; 10 Department of Hematology, Zealand University Hospital, Roskilde, Denmark; 11 Department of Hematology, Vejle Sygehus, Vejle, Denmark; 12 Department of Hematology, Sydvestjysk sygehus, Esbjerg, Denmark; 13 Department of Medicine, Holstebro sygehus, Holstebro, Denmark; 14 Department of Cardiology, Nordsjællands Hospital, Hillerød, Denmark; 15 Department of Public Health, University of Copenhagen, Copenhagen, Denmark

**Keywords:** B-cell lymphoma, Cancer epidemiology

## Abstract

For most patients with newly diagnosed diffuse large B-cell lymphoma (DLBCL), R-CHOP immunochemotherapy leads to complete remission and 60–70% of patients remain progression-free after 5 years. Given a median age of 65, it is relevant to disentangle how DLBCL and DLBCL therapy influence health care use among the survivors. In this nationwide study, the health care use among Danish DLBCL patients diagnosed in 2007–2015, who achieved complete remission after R-CHOP(-like) therapy, was explored and compared to matched comparators from the Danish general population. The post-remission 5-year risk of hospitalization was significantly higher among DLBCL survivors (55%) compared to matched comparators (49%, *P* < 0.001). DLBCL survivors had on average 10.3 (9.3–11.3) inpatient bed days within 5 years of response evaluation, whereas matched comparators had 8.4 (7.9–8.8). The rate of outpatient visits was also significantly higher(excluding routine follow-up visits, incidence rate ratio, 1.3, *P* < 0.001), but translated into only a very small absolute difference of <1 outpatient visits within 5 years between DLBCL survivors (4.2 visits, 95% CI, 4.0–4.4) and matched comparators (3.8 visits, 95% CI, 3.7–3.9). In conclusion, DLBCL survivors have an increased incidence of hospital visits due to a wide range of conditions, but in absolute terms the excess use of health care services in DLBCL survivors was small.

## Introduction

Diffuse large B-cell lymphoma (DLBCL) is the most common type of non-Hodgkin lymphoma in the western world with an annual incidence of 7 per 100,000 person-years in the United States [1]. The likelihood of achieving complete remission (CR) after first-line rituximab, cyclophosphamide, doxorubicin, vincristine, and prednisolone (R-CHOP) treatment is ≥75% for patients who tolerate the standard six cycles [2, 3]. Patients in first CR have good survival outcomes compared to many other cancer patients with the risk of relapse during the first 5 years being 18% and as low as 8% for patients without progression during the first 2 years following response evaluation [4]. This means that the majority of DLBCL patients in first CR can be considered cured of their lymphoma. This is supported by recent studies showing that the mortality of DLBCL patients, who remain event-free, approximates that of the general population only a few years after diagnosis [4, 5]. As the median age of DLBCL patients in CR after R-CHOP(-like) therapy is 65 years [4], the average DLBCL survivor will face a residual lifetime of >10 years. Patients will be at risk of developing late adverse complications to the R-CHOP treatment during these years, with the most well-described being secondary malignancies and cardiotoxicity [6, 7]. Except for the most common early adverse events, comprehensive safety assessments are notoriously difficult to perform in clinical trials, as these are often underpowered to detect infrequent safety signals and have a too short follow-up to assess long-term adverse events. Although observational studies have an advantage in size and follow-up, these studies typically focus on one or a few specific late complications and not the overall health care use among DLBCL survivors, which can be considered a surrogate for overall health [8]. This nationwide register study investigated the use of health care services among Danish DLBCL survivors by quantifying the risk of hospitalization as well as the rate of inpatient bed days and outpatient visits in comparison to that of a Danish matched general population.

## Methods and patients

### Patients and comparators

Newly diagnosed Danish DLBCL patients were identified from the Danish National Lymphoma Registry (LYFO), which covers ~95% of all lymphoma patients in Denmark [9]. For each patient, the registry captures extensive information on clinicopathological characteristics, treatment, treatment response, relapse, and death. Details on LYFO can be found elsewhere [10]. Patients diagnosed between 2007 and 2015 were included if they met the following criteria: (1) ≥18 years of age at diagnosis, (2) treated with 6–8 cycles of R-CHOP or R-CHOEP in the first line, (3) in CR/CR unconfirmed (CRu) after first-line treatment according to PET or CT-based response criteria, and (4) alive and relapse-free 90 days after the end of treatment response evaluation. The latter inclusion criterium was used to minimize the number of hospital contacts due to early relapses. Patients with primary CNS lymphoma, CNS involvement at the time of diagnosis, discordant/composite lymphoma, or Richter transformation were excluded. The included patients will be coined “survivors” in the remaining.

For each DLBCL survivor, five comparators from the Danish general population were identified from the Danish Civil Registration System, which contains information from all Danish citizens on vital status, emigration, and other personal information [11]. Matching was performed on sex, birth year, and Charlson comorbidity index [12] (CCI, at 180 days before the date of DLBCL diagnosis). Comparators had to be alive without a previous lymphoma diagnosis and living in Denmark on the 90th day after response evaluation for the index survivor.

### Hospital visits

Outpatient visits to specialized clinics and hospital admissions including duration were identified through queries to the Danish National Patient Register (DNPR), which covers all visits to public hospitals in Denmark. Since 2003, it has also been mandatory for private health care providers to report activities to the DNPR, but ambulatory visits to private practice specialists and general practitioners are not registered [13]. From 1994 onwards, all diagnoses have been classified according to ICD-10. Visits were grouped according to ICD-10 chapters similarly to the methodology of a previous Hodgkin lymphoma study (Table S1) [14], and separate analyses were conducted for outpatient visits and inpatient bed days. For ICD-10 chapters where rates differed significantly between DLBCL survivors and matched comparators, the five most frequent sub-diagnoses in the DLBCL survivors were identified. Sub-diagnoses within the ICD-10 chapters were grouped using only the first two digits of the ICD-10 codes (e.g., A00). Admissions to intensive care units (ICUs) were identified by queries to the DNPR using the Danish procedure codes NABB and NABE [15]. For all in- and outpatient hospital contacts, the primary diagnosis was considered the reason for the contact, and secondary diagnoses were not considered.

As the primary focus of this study was to investigate the potential excess health care use among DLBCL survivors related to health problems and not routine lymphoma surveillance, outpatient visits with lymphoma (including DLBCL and other lymphomas) as the primary diagnosis were disregarded. The Charlson comorbidity index was computed based on diagnoses registered in association with hospital visits as well as drug prescriptions in the Danish National Prescription Registry, which covers prescription drugs sold in Danish community pharmacies [16]. Vital status (including information on emigration) for survivors and comparators was obtained from the Danish Civil Registration System [11].

### Statistics

Post-remission overall survival (pOS) was measured from 90 days after final response evaluation (index date) until death or censoring (including emigration and administrative censoring in December 2017). Survival curves were computed using the Kaplan–Meier estimator and differences were tested using the log-rank test. For all other analyses, follow-up was terminated 10 years after the index date or at the time of relapse in DLBCL survivors who eventually relapsed. The Aalen–Johansen estimator was used to compute cumulative risks of hospitalization treating death and lymphoma relapse as competing events. For matched comparators who later developed DLBCL, the DLBCL diagnosis was also considered a competing event. Differences in overall risks were tested using Grays’ test [17], while differences in 5-year risks were tested using the pseudo-observation approach described by Andersen and Perme [18]. Incidence rates (IRs) for hospital visits were computed for both DLBCL survivors and comparators and IR ratios (IRRs) were calculated by Poisson regression with the logarithm of person-time as offset [19]. The mean number of inpatient bed days within 5 years of index date was calculated separately for DLBCL survivors and matched comparators by plugging the marginal hazard rates for inpatient bed days and competing events (death, relapse, and diagnosis of lymphoma [in matched comparators]) into the formula for the marginal mean number of events described by Ghosh and Lin [20]. Confidence intervals for the marginal mean number of events were computed by nonparametric bootstrap using 1000 resamples. A similar analysis was conducted for outpatient visits. Landmark analyses were conducted by including only DLBCL survivors who survived two and 5 years without experiencing a relapse. At each landmark time point, five new comparators were matched to the survivors, and rates were computed for the time periods 0–2, 2–5, and 5–10 years.

## Results

### Characteristics of DLBCL survivors and post-remission hospitalization

**Fig. 1:**
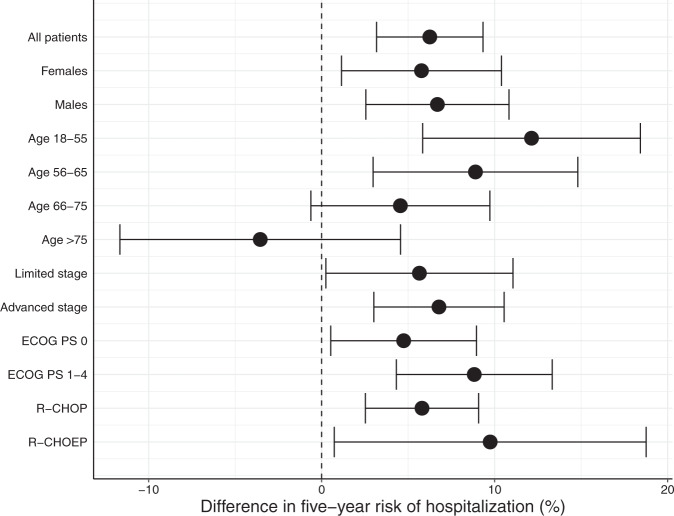
Differences in post-remission 5-year risk of hospitalization between DLBCL survivors and matched comparators from the Danish general population. The differences are reported overall and within clinical subgroups. Elevated risks of hospitalization among DLBCL survivors correspond to a positive risk difference and error bars indicate 95% confidence intervals. ECOG PS Eastern Cooperative Oncology Group performance status.

In total, 1446 DLBCL survivors and 7230 matched comparators were included in the study (Table 1). With a median follow-up of 64 months, the 5-year pOS was 81% (95% CI, 79–83%) and 89% (95% CI, 88–90%) for DLBCL survivors and matched comparators, respectively (*P* < 0.001, Fig. S1). The 5-year risk of hospitalization was 55% (95% CI, 52–58%) among the survivors and 49% (95% CI, 47–50%) among matched comparators (*P* < 0.001, Fig. S2). The 5-year risk of hospitalization was significantly elevated within the majority of clinical subgroups, particularly among those youngest at diagnosis (Fig. 1). Within subgroups with significantly elevated 5-year risk of hospitalization, the absolute risk differences ranged from 5% (among survivors with performance score 0 at diagnosis) to 12% (among survivors aged 18–55 years). The 5-year risk of hospitalization was 56% (95% CI, 53–59%) and 44% (95% CI, 36–52%) for survivors treated with CHOP and CHOEP, respectively. When compared to the 5-year risk of hospitalization among matched comparators, this translated into 6 and 10% absolute differences (relative differences of 11 and 29%) for CHOP and CHOEP-treated patients, respectively (Fig. 1). The risk of critical illness, defined by ICU admission, was not significantly higher among DLBCL survivors (*P* = 0.073, Fig. S3), and the absolute difference in 5-year risk of ICU admission between DLBCL survivors and matched comparators was −0.07% (DLBCL survivors: 4.97%, matched comparators: 5.04%).

**Table 1:** Diagnostic clinicopathological characteristics of all Danish DLBCL patients in CR after 6–8 cycles of R-CHOP-like therapy and free of relapse 90 days after response evaluation.

	DLBCL survivors (*n* = 1446)	Comparators (*n* = 7230)
Male, *n*(%)	798 (55.2)	3990 (55.2)
Median age (range)	66 (18–89)	66 (18–90)
Age groups, *n*(%)		
- 18–55	326 (22.5)	1573 (21.8)
- 56–65	367 (25.4)	1830 (25.3)
- 66–75	523 (36.2)	2614 (36.2)
- >75	230 (15.9)	1213 (16.8)
Charlson comorbidity index, *n*(%)		
- 0	829 (57.3)	4145 (57.3)
- 1	314 (21.7)	1570 (21.7)
- 2	170 (11.8)	850 (11.8)
- >2	133 (9.2)	665 (9.2)
Ann Arbor stage III–IV, *n*(%)	953 (66.3)	
Performance status >0, *n*(%)	656 (45.6)	
Extranodal involvement, *n*(%)	890 (61.5)	
Elevated LDH, *n*(%)	804 (56.8)	
B symptoms, *n*(%)	631 (44.5)	
Chemotherapy, *n*(%)		
- R-CHOEP	171 (11.8)	
- R-CHOP	1275 (88.2)	
Cycles, *n*(%)		
- 6	1156 (79.9)	
- 7–8	290 (20.1)	
Radiotherapy, *n*(%)	341 (23.6)	

The Charlson comorbidity index was evaluated 180 days before the DLBCL diagnosis.

*LDH* lactate dehydrogenase.

### Inpatient bed days

**Fig. 2:**
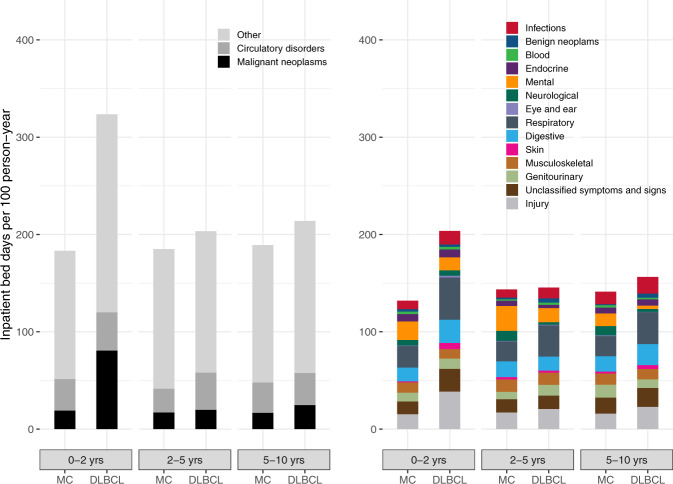
Incidence rates of inpatient bed days stratified by ICD-10 chapter among survivors and matched comparators in different time periods (0–2, 2–5, 5–10 years after the index date). The incidence rates of conditions included in the “Other” category in the left-hand panel are displayed in the right-hand panel. DLBCL diffuse large B-cell lymphoma, MC matched comparators.

The mean number of bed days within 5 years was significantly higher among DLBCL survivors (10.3, 95% CI 9.3–11.3) compared to matched comparators (8.4, 95% CI 7.9–8.8), with an absolute difference of 1.9 days (Table 2). DLBCL survivors had a significantly increased rate of inpatient bed days within each ICD-10 chapter (except mental, genitourinary, endocrine, neurological, blood, and eye and ear conditions, Table S2). The most common reasons for hospitalization in DLBCL survivors were malignant neoplasms, circulatory disorders, and respiratory conditions (Fig. 2). For malignant neoplasm, the IR was 45.7 and 16.7 days/100 person-years for DLBCL survivors and comparators, respectively, (IRR, 2.73, *P* < 0.001, Table S2), whereas for circulatory disorders the IR was 37.5 and 29.6 days/100 person-years for DLBCL survivors and comparators, respectively (IRR, 1.27, *P* < 0.001, Table S2). For respiratory conditions, the IR was 36.9 days/100 person-years for DLBCL survivors and 22.2 for matched comparators (IRR, 1.67, *P* < 0.001). The main driver of visits due to malignant neoplasms was lymphoma-related bed days followed by malignant neoplasms of bronchus and lung, and secondary malignant neoplasm of respiratory and digestive organs (Table S3). Within circulatory disorders, heart failure, atrial fibrillation, and cerebral infarction were the main drivers of inpatient bed days, while pneumonia (organism unspecified), respiratory failure, and bacterial pneumonia were the main drivers within respiratory conditions (Table S3). The IRR of inpatient bed days due to malignant neoplasms decreased over time but remained significantly different from one in all time periods (Table S4).

**Table 2:** The mean number of inpatient bed days and outpatient visits within 5 years of the index date (date of response evaluation + 90 days) for DLBCL survivors and matched comparators without lymphoma.

		Mean number of bed days		Mean number of outpatient visits	
	DLBCL survivors	DLBCL survivors	Comparators	DLBCL survivors	Comparators
All patients	1446	10.3 (9.3–11.3)	8.4 (7.9–8.8)	4.2 (4.0–4.4)	3.8 (3.7–3.9)
Sex					
Females	648	9.9 (8.5–11.2)	7.7 (7.1–8.3)	4.3 (4.0–4.6)	3.8 (3.6–3.9)
Males	798	10.7 (9.3–12.0)	8.9 (8.3–9.5)	4.1 (3.8–4.3)	3.8 (3.7–3.9)
Age					
18–55	326	6.2 (4.5–7.7)	4.4 (3.4–5.2)	3.5 (3.1–3.8)	2.6 (2.4–2.7)
56–65	367	10.2 (8.1–12.1)	7.2 (6.4–8.0)	3.9 (3.6–4.3)	3.3 (3.2–3.4)
66–75	523	11.4 (10.0–12.9)	9.7 (9.0–10.4)	4.7 (4.3–5.0)	4.6 (4.4–4.7)
>75	230	14.8 (11.3–17.8)	13.9 (12.8–14.8)	4.8 (4.2–5.3)	4.7 (4.5–5.0)
Stage					
Limited (I–II)	485	9.5 (7.7–11.1)	8.2 (7.4–9.0)	4.3 (4.0–4.6)	3.7 (3.6–3.9)
Advanced (III–IV)	953	10.8 (9.5–12.0)	8.4 (7.9–8.9)	4.2 (3.9–4.4)	3.8 (3.7–3.9)
ECOG PS					
0	783	8.7 (7.3–9.9)	7.5 (6.9–8.0)	4.0 (3.8–4.3)	3.6 (3.5–3.7)
1–4	656	12.4 (10.9–13.7)	9.1 (8.5–9.8)	4.4 (4.2–4.7)	4.0 (3.8–4.1)

For estimates within specific clinical subgroups (e.g., limited-stage disease), estimates for comparators were only based on comparators matched to the patients in the subgroup.

*ECOG PS* Eastern Cooperative Oncology Group performance status, *LDH* lactate dehydrogenase.

### Outpatient visits

**Fig. 3:**
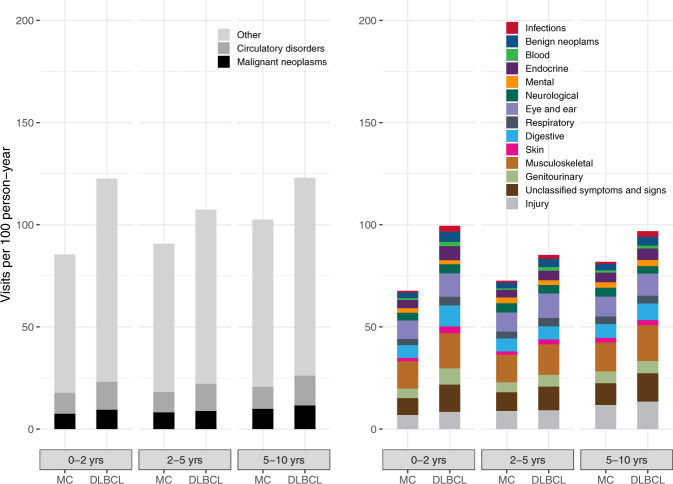
Incidence rates of hospital outpatient specialist clinic visits stratified by ICD-10 chapter among survivors and matched comparators in different time periods (0–2, 2–5, 5–10 years after the index date). The incidence rates of conditions included in the “Other” category in the left-hand panel are displayed in the right-hand panel. DLBCL diffuse large B-cell lymphoma, MC matched comparators.

The mean number of outpatient visits (excluding lymphoma visits) within 5 years was 4.2 (95% CI, 4.0–4.4) among DLBCL survivors and 3.8 (95% CI, 3.7–3.9) in matched comparators. The excess number of outpatient visits within 5 years, i.e., the difference between the number of visits for DLBCL survivors and their comparators, was ≤1 within all clinical subgroups (Table 2). However, the IRR for outpatient visits was 1.3 (95% CI, 1.2–1.3, *P* < 0.001), and for the majority of ICD-10 chapters, DLBCL survivors had a significantly increased rate of outpatient visits (Table S5). The most common reasons for outpatient visits in DLBCL survivors were musculoskeletal conditions, unclassified symptoms and signs, and circulatory disorders (Fig. 3). For musculoskeletal conditions, the IR was 14.6 and 12.5 visits/100 person-years for DLBCL survivors and comparators, respectively (IRR, 1.17, *P* < 0.001, Table S5), whereas for unclassified symptoms and signs (ICD-10 codes R00-R99), the IR for survivors and matched comparators was 11.9 and 8.4 visits/100 person-years, respectively, (IRR, 1.41, *P* < 0.001). For circulatory conditions, the IR was 11.9 and 9.4 for survivors and matched comparators, respectively (IRR, 1.27, *P* < 0.001). The main driver of outpatient visits due to musculoskeletal conditions were arthrosis of the hip, osteoporosis without pathological fracture, and arthrosis of the knee, although outpatient visits due to arthrosis of the knee were not significantly more frequent in DLBCL survivors (Table S6). Within unclassified symptoms and signs the main drivers were abdominal and pelvic pain, abnormalities of breathing, and abnormal findings on diagnostic imaging of lung, while for circulatory conditions, the main drivers were atrial fibrillation and flutter, heart failure, and non-rheumatic aortic valve disorders (Table S6). Generally, the IRR of outpatient visits in each ICD-10 chapter decreased over time and approached one (Table S7), pointing towards less differences between DLBCL survivors and matched comparators as time elapsed without relapse. However, for 5/16 ICD-10 chapters (including infections and circulatory disorders), the rate of outpatient visits was still significantly elevated in the period 5–10 years (Table S7).

## Discussion

In this nationwide study, we investigated the use of health care services among Danish DLBCL survivors in CR after first-line treatment with 6–8 cycles of R-CHOP(-like) immunochemotherapy. Findings were compared to matched individuals from the Danish general population with the same age, sex, and comorbidity index. We observed an increased risk of hospitalization among DLBCL survivors, but the differences were limited with DLBCL survivors having 1.9 inpatient bed day more than matched comparators during the 5 years following the index date. Surprisingly, the risk of hospitalization was not elevated among survivors >75 years of age at diagnosis, likely because these elderly survivors eligible for 6–8 cycles of R-CHOP constitute a highly selected fit group, making them healthier than the elderly general population. Although the rate of outpatient visits differed significantly between DLBCL survivors and matched comparators overall (IRR, 1.3) and within many of the considered ICD-10 chapters, this translated into only a very small difference in the mean number of outpatient visits within 5 years. The very small differences are surprising and could reflect limited complications among DLBCL survivors or that routine lymphoma visits are sometimes also used to address other health problems that are manageable without additional visits. Importantly, we only considered visits that occurred >90 days after the response evaluation excluding findings of medical conditions or complications directly after immunochemotherapy. The higher event (death and relapse) rate among DLBCL survivors may also reduce differences between the survivors and the matched comparators in analyses of the mean number of visits as these take competing events into account. To investigate the difference in a population not affected by differences in relapse or death, we conducted a sensitivity analysis including only survivors and comparators with complete follow-up and no competing events within the first 5 years following the index date and computed the average number of bed days and outpatient visits during these 5 years. This analysis confirmed the limited difference in the average number of inpatient bed days (absolute difference, 2.1 days) and outpatient visits (absolute difference, 0.9 visits) between DLBCL survivors and matched comparators.

To our knowledge, this is the first study to highlight the overall health care use of DLBCL survivors in a population-based setting. However, this has previously been investigated for Hodgkin lymphoma (HL) survivors in a Swedish nationwide study [14]. Consistent with the HL study, DLBCL survivors in the present study had an elevated number of inpatient bed days and an increased rate of outpatient visits as compared to the general population. However, the absolute excess number of outpatient visits was smaller in our study compared to the HL study. Differences between DLBCL survivors and matched comparators are likely driven by multiple factors. Health problems caused by late toxicities after R-CHOP is an obvious explanation, but it could also be at least partially explained by surveillance bias since lymphoma survivors are screened regularly for lymphoma relapse, sometimes including imaging, which may lead to clinical findings that would otherwise have remained undiscovered. Regardless of the reasons, the absolute differences in the mean number of inpatient bed days and outpatient visits were small to very modest at best in this analysis of >8000 individuals. This is important, as DLBCL survivors and their relatives can be informed of a generally good health prognosis besides the already known limited risk of lymphoma relapse.

In the present study, we observed increased hospital visits within areas where it has been shown that DLBCL therapy are associated with late complications. The incidence of musculoskeletal conditions was slightly elevated in DLBCL survivors, which is in line with our previous results showing an elevated risk of osteoporotic events in patients with non-Hodgkin lymphoma [21]. For neurological and mental disorders, we did not observe a higher rate of neither inpatient bed days nor outpatient visits. A diagnosis of lymphoma (or other cancer types) is associated with mental stress, which in some cases requires treatment for depression or anxiety. This has been highlighted by the increased use of psychotropic drugs among newly diagnosed HL patients when compared to the general population [22]. Since the vast majority of mental disorders are often treated in primary care, the present study would likely not capture the true incidence of mental disorders in DLBCL survivors.

Cardiovascular toxicity is a well-known side effect of doxorubicin [7, 23]. All patients in the present study were treated with 6–8 cycles of R-CHOP or R-CHOEP in Denmark, where the standard dosage of doxorubicin is 50 mg/m^2^ per cycle. In the present study, we observed an increased rate of inpatient bed days and outpatient visits due to circulatory disorders. This has also been observed among HL patients in the Swedish study, but in that study, the rate >6 years after diagnosis was no longer significantly different from the rate among matched comparators without HL [14]. In the present study, we only observed a minor increase in the rate of outpatient visits due to circulatory disorders after 5 years (Table S7). There are important differences in the cause of cardiotoxicity between HL and DLBCL survivors. HL survivors will more often have received radiotherapy for mediastinal tumors, which comes with an increased risk of coronary or valvular heart disease, congestive heart failure, and pericarditis [24]. We have previously shown that the use of anthracycline-containing chemotherapy in patients with non-Hodgkin lymphoma increases the risk of congestive heart failure and cardiovascular disease and that there is a dose-response relationship between anthracycline and cardiotoxicity [7]. This is also indicated by an increased risk of cardiac adverse events among patients treated with 8 cycles of CHOP compared to six cycles in the GOYA trial [25]. Screening of elderly patients for congestive heart failure prior to commencing R-CHOP therapy may have reduced the differences between DLBCL survivors and comparators in terms of visits related to cardiovascular diagnoses. Similar screening would not have been performed in the general population and therefore individuals with silent congestive heart failure would not have been identified and excluded in the same manner.

Secondary malignancies are a major concern in successfully treated DLBCL patients. Consistently with existing literature, we found an excess rate of inpatient bed days and outpatient visits due to malignant neoplasms among the DLBCL survivors [6]. Malignant neoplasms of bronchus and lung, secondary malignant neoplasm of respiratory and digestive organs, and secondary malignant neoplasm of other and unspecified sites, which covers metastasized malignant neoplasms, were the main drivers (besides non-Hodgkin lymphoma) of inpatient bed days due to malignant neoplasms (Table S3). However, the IRR of inpatient bed days due to malignant neoplasms was reduced substantially after 2 years but remained significantly different from one after this time point. To examine the effect of increased examinations prior to relapse confirmation on the rate of visits due to non-Hodgkin lymphoma, we conducted a sensitivity analysis where relapses were brought forward by 30 days. In this analysis, the IR for inpatient bed days due to non-follicular lymphoma changed from 22.7 (Table S3) to 17.8, but this remained the main driver of inpatient bed days due to malignant neoplasms (data not shown), suggesting that the excess rate of lymphoma inpatient bed days was not primarily due to examinations on the ground of relapse suspicions.

The strengths of the present study include the nationwide data from a country with universal access to health care, which minimizes selection bias that affects the generalizability of the results. Additionally, extensive clinical and historical patient-level data were obtained by merging with nationwide registers including information on all hospital visits, pathology results, and ICU admissions. Using the detailed information from LYFO, we were able to extract a homogeneous cohort of patients responding to standard R-CHOP(-like) therapy, a cohort in which concerns of adverse late effects are particularly relevant. The study had a limited median follow-up of 5 years and hence the incidence of very late adverse events in DLBCL survivors was not investigated. Furthermore, our analyses relied exclusively on the primary diagnoses, which may not always reflect the exact reason for hospital visits. We excluded outpatient visits for which the primary diagnosis was lymphoma, as these were interpreted as related to lymphoma surveillance, but it is possible that this diagnosis was mistakenly assigned by other specialists if a non-lymphoma-related outpatient visit did not result in a specific primary diagnosis. This would lead to underestimation of outpatient visits among the DLBCL survivors. However, in a sensitivity analysis removing only lymphoma outpatient visits registered at hematology departments, the average number of outpatient visits within 5 years only increased by 0.2 for DLBCL survivors. Thus, differences between DLBCL survivors and comparators in terms of outpatient activities remained minor. Some hospital outpatient contacts registered in DNPR may cover more than one visit at the same department and were registered to last more than 1 day. Thus, the number of hospital outpatient visits observed in the present study may be slightly underestimated. However, the proportion of all outpatient visits extending 30 days (i.e., longer ambulatory contacts likely with more than one actual visit at the department) was balanced between the DLBCL survivors (51%) and matched comparators (50%). Due to data limitations, the study is not able to inform whether health problems are generally more severe in lymphoma survivors than in matched comparators. The fact that the differences in hospital bed days and ICU admissions were only minor are not necessarily evidence for the absence of increased health problems in DLBCL survivors. Many symptoms and conditions related to late toxicities can often be managed in primary care or as part of routine lymphoma follow-up. Thus, referrals and hospital admissions are only relevant in the most severe cases or cases that require diagnostic work-up or treatments by other hospital specialists.

In conclusion, DLBCL survivors face an elevated rate of a wide range of medical conditions highlighting the importance of broad follow-up programs that also focus on late toxicities. Reassuringly to future DLBCL survivors, the differences between DLBCL survivors and their matched comparators in terms of a mean number of inpatient bed days and outpatient visits were small and with limited clinical significance on an individual patient-level during the first 5 years of follow-up.

## Supplementary information

Supplementary material

Aj checklist

## Data Availability

All analyses were conducted in SAS (SAS Institute Inc., Gary, NC, USA) and R (version 4.0.3). Programs for running the analysis are not publicly available but can be provided by the corresponding author per request. The study was approved by the Danish Data Protection Agency (2008-58-0028).

## References

[1] Li Y, Wang Y, Wang Z, Yi D, Ma S (2015). Racial differences in three major NHL subtypes: descriptive epidemiology. Cancer Epidemiol.

[2] Coiffier B, Thieblemont C, Van Den Neste E, Lepeu G, Plantier I, Castaigne S (2010). Long-term outcome of patients in the LNH-98.5 trial, the first randomized study comparing rituximab-CHOP to standard CHOP chemotherapy in DLBCL patients: a study by the Groupe d’Etudes des Lymphomes de l’Adulte. Blood.

[3] Pfreundschuh M, Schubert J, Ziepert M, Schmits R, Mohren M, Lengfelder E (2008). Six versus eight cycles of bi-weekly CHOP-14 with or without rituximab in elderly patients with aggressive CD20+ B-cell lymphomas: a randomised controlled trial (RICOVER-60). Lancet Oncol.

[4] Jakobsen LH, Bøgsted M, Brown P, de N, Arboe B, Jørgensen J (2017). Minimal loss of lifetime for patients with diffuse large B-cell lymphoma in remission and event free 24 months after treatment: a Danish population-based study. J Clin Oncol.

[5] Maurer MJ, Ghesquières H, Jais J-P, Witzig TE, Haioun C, Thompson CA (2014). Event-free survival at 24 months is a robust end point for disease-related outcome in diffuse large B-cell lymphoma treated with immunochemotherapy. J Clin Oncol.

[6] Tao L, Clarke CA, Rosenberg AS, Advani RH, Jonas BA, Flowers CR (2017). Subsequent primary malignancies after diffuse large B-cell lymphoma in the modern treatment era. Br J Haematol.

[7] Baech J, Hansen SM, Lund PE, Soegaard P, Brown P, de N (2018). Cumulative anthracycline exposure and risk of cardiotoxicity; a Danish nationwide cohort study of 2440 lymphoma patients treated with or without anthracyclines. Br J Haematol.

[8] Thanarajasingam G, Minasian LM, Baron F, Cavalli F, De Claro RA, Dueck AC (2018). Beyond maximum grade: modernising the assessment and reporting of adverse events in haematological malignancies. Lancet Haematol.

[9] Arboe B, El-Galaly TC, Clausen MR, Munksgaard PS, Stoltenberg D, Nygaard MK, et al. The Danish National Lymphoma Registry: coverage and data quality. PLoS ONE. 2016;11:e0157999. 10.1371/journal.pone.0157999 PMC4919044 27336800

[10] Arboe B, Josefsson P, Jørgensen J, Haaber J, Jensen P, Poulsen C (2016). Danish National Lymphoma Registry. Clin Epidemiol.

[11] Schmidt M, Pedersen L, Sørensen HT (2014). The Danish civil registration system as a tool in epidemiology. Eur J Epidemiol.

[12] Charlson M, Szatrowski TP, Peterson J, Gold J (1994). Validation of a combined comorbidity index. J Clin Epidemiol.

[13] Schmidt M, Schmidt SAJ, Sandegaard JL, Ehrenstein V, Pedersen L, Sørensen HT (2015). The Danish National patient registry: a review of content, data quality, and research potential. Clin Epidemiol.

[14] Glimelius I, Eloranta S, Ekberg S, Chang ET, Neovius M, Smedby KE (2017). Increased healthcare use up to 10 years among relapse‐free Hodgkin lymphoma survivors in the era of intensified chemotherapy and limited radiotherapy. Am J Hematol.

[15] Christiansen CF, Møller MH, Nielsen H, Christensen S (2016). The Danish intensive care database. Clin Epidemiol.

[16] Wallach Kildemoes H, Toft Sørensen H, Hallas J (2011). The Danish National Prescription Registry. Scand J Public Health.

[17] Gray RJ (1988). A class of K-sample tests for comparing the cumulative incidence of a competing risk on JSTOR. Ann Stat.

[18] Andersen PK, Pohar Perme M (2010). Pseudo-observations in survival analysis. Stat Methods Med Res.

[19] Lawless JF, Crowder MJ (2010). Models and estimation for systems with recurrent events and usage processes. Lifetime Data Anal.

[20] Ghosh D, Lin DY (2000). Nonparametric analysis of recurrent events and death. Biometrics.

[21] Baech J, Hansen SM, Jakobsen LH, Øvlisen AK, Severinsen MT, Brown P (2020). Increased risk of osteoporosis following commonly used first-line treatments for lymphoma: a Danish Nationwide Cohort Study. Leuk Lymphoma.

[22] Øvlisen AK, Jakobsen LH, Kragholm KH, Nielsen RE, Hutchings M, Dahl‐Sørensen RB, et al. Depression and anxiety in Hodgkin lymphoma patients: a Danish nationwide cohort study of 945 patients. Cancer Med. 2020;9:4395–404. 10.1002/cam4.2981 PMC7300408 32301251

[23] Lee SF, Luque-Fernandez MA, Chen YH, Catalano PJ, Chiang CL, Wan EYF (2020). Doxorubicin and subsequent risk of cardiovascular diseases among survivors of diffuse large B-cell lymphoma in Hong Kong. Blood Adv.

[24] Van Leeuwen FE, Ng AK (2016). Long-term risk of second malignancy and cardiovascular disease after Hodgkin lymphoma treatment. Hematology.

[25] Sehn LH, Congiu AG, Culligan DJ, Gironella M, Yoon DH, Ogura M (2018). No added benefit of eight versus six cycles of CHOP when combined with rituximab in previously untreated diffuse large B-cell lymphoma patients: results from the international phase III GOYA study. Blood.

