# Supplementary material for: Patients in complete remission after R-CHOP(-like) therapy for diffuse large B-cell lymphoma have limited excess use of health care services in Denmark
Source: Blood Cancer J. 2022 Jan 27;12(1):16. doi: 10.1038/s41408-022-00614-8 (PMC8795387; doi:10.1038/s41408-022-00614-8)
Supplement: Supplementary file 1 — Supplementary material [file 41408_2022_614_MOESM1_ESM.docx]

Supplementary

Figures

Figure S1: Post-remission overall survival for 1,446 DLBCL survivors in complete remission following first-line R-CHOP-like chemotherapy and 7,230 matched comparators. The shaded areas indicate 95% confidence intervals.
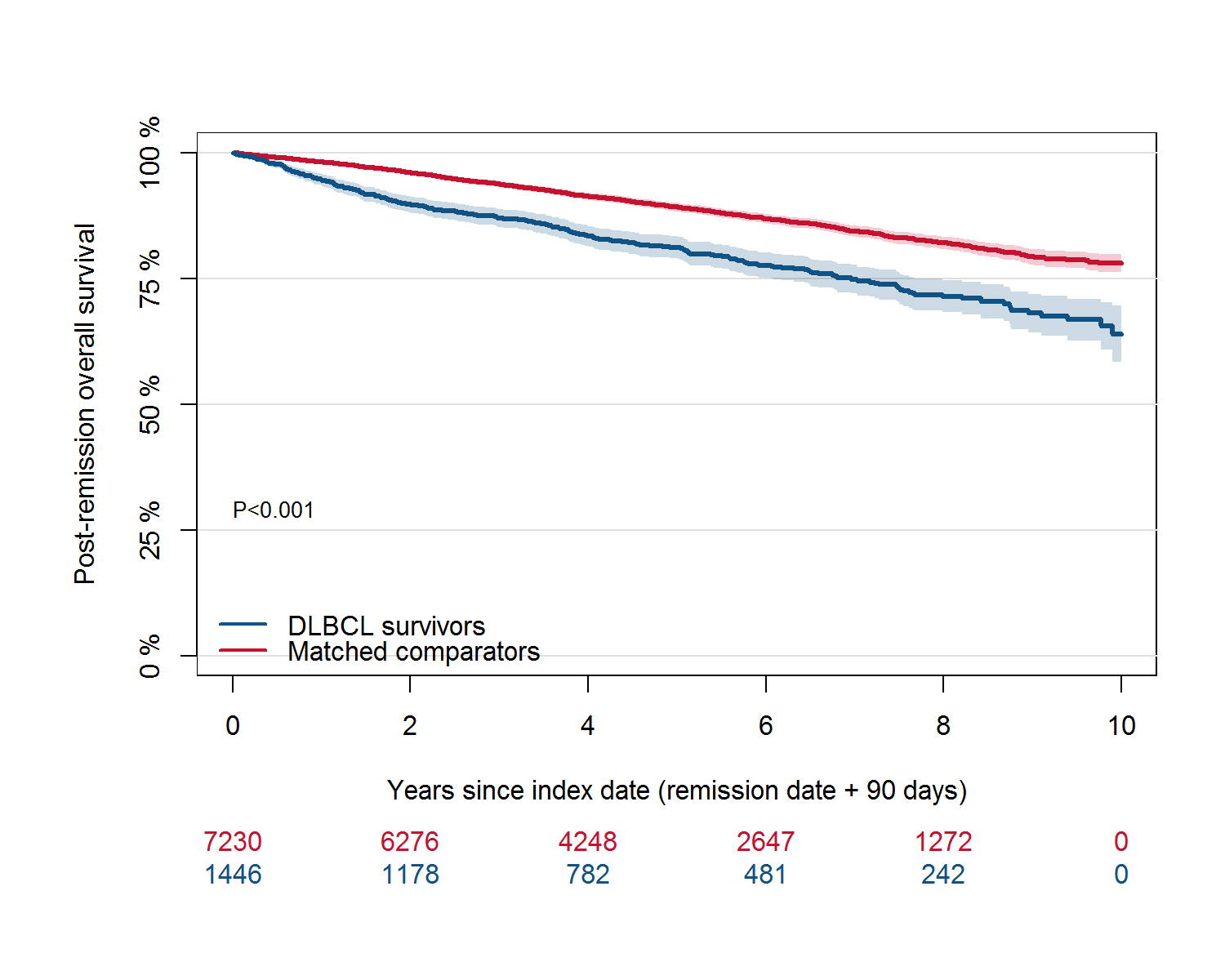


Figure S2: The cumulative risk of post-remission hospitalization for 1,446 DLBCL survivors in complete remission following first-line R-CHOP-like chemotherapy and 7,230 matched comparators. The shaded areas indicate 95% confidence intervals


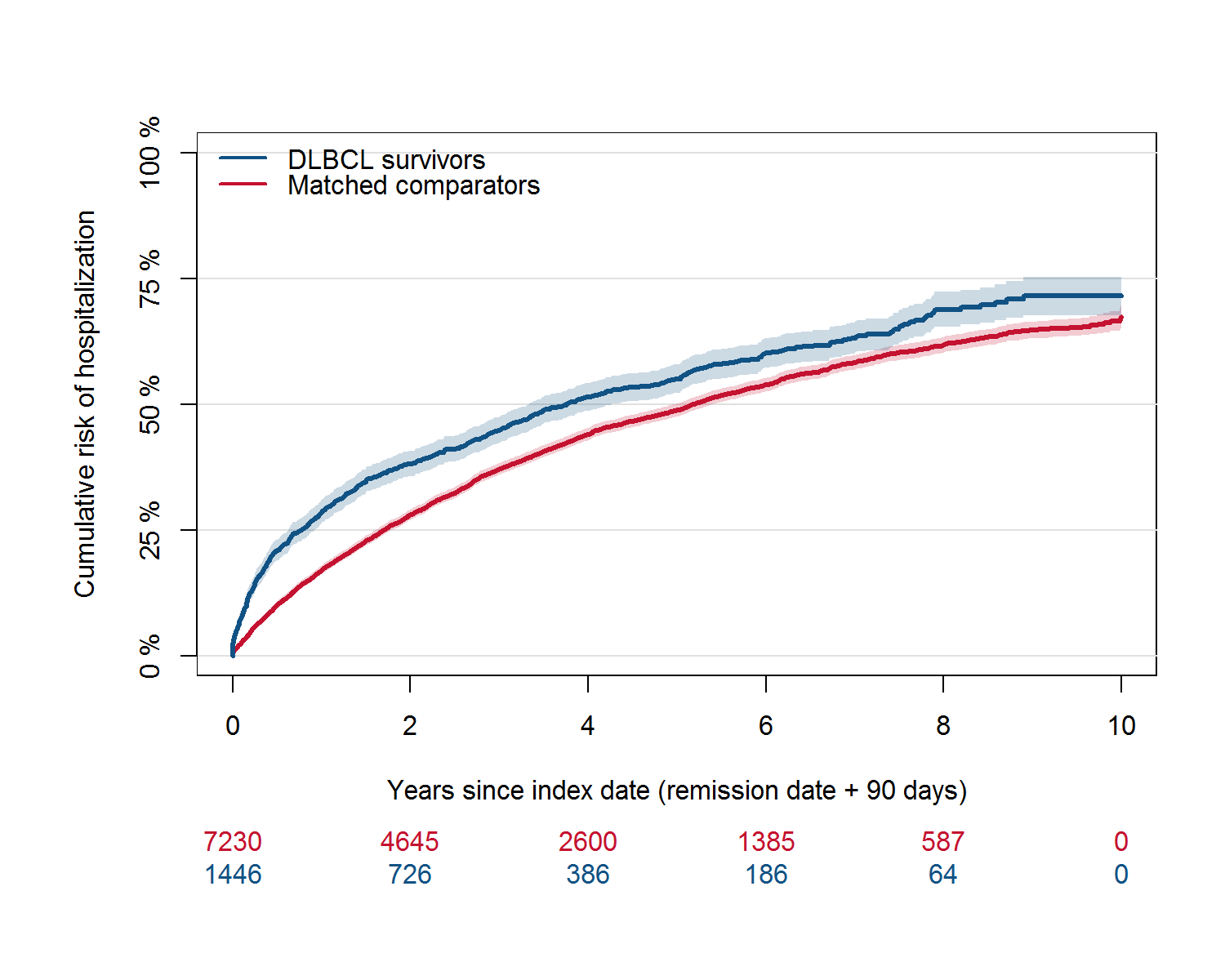


Figure S3: The cumulative risk of admission to intensive care unit (ICU) for 1,446 DLBCL survivors in complete remission following first-line R-CHOP-like chemotherapy and 7,230 matched comparators. The shaded areas indicate 95% confidence intervals
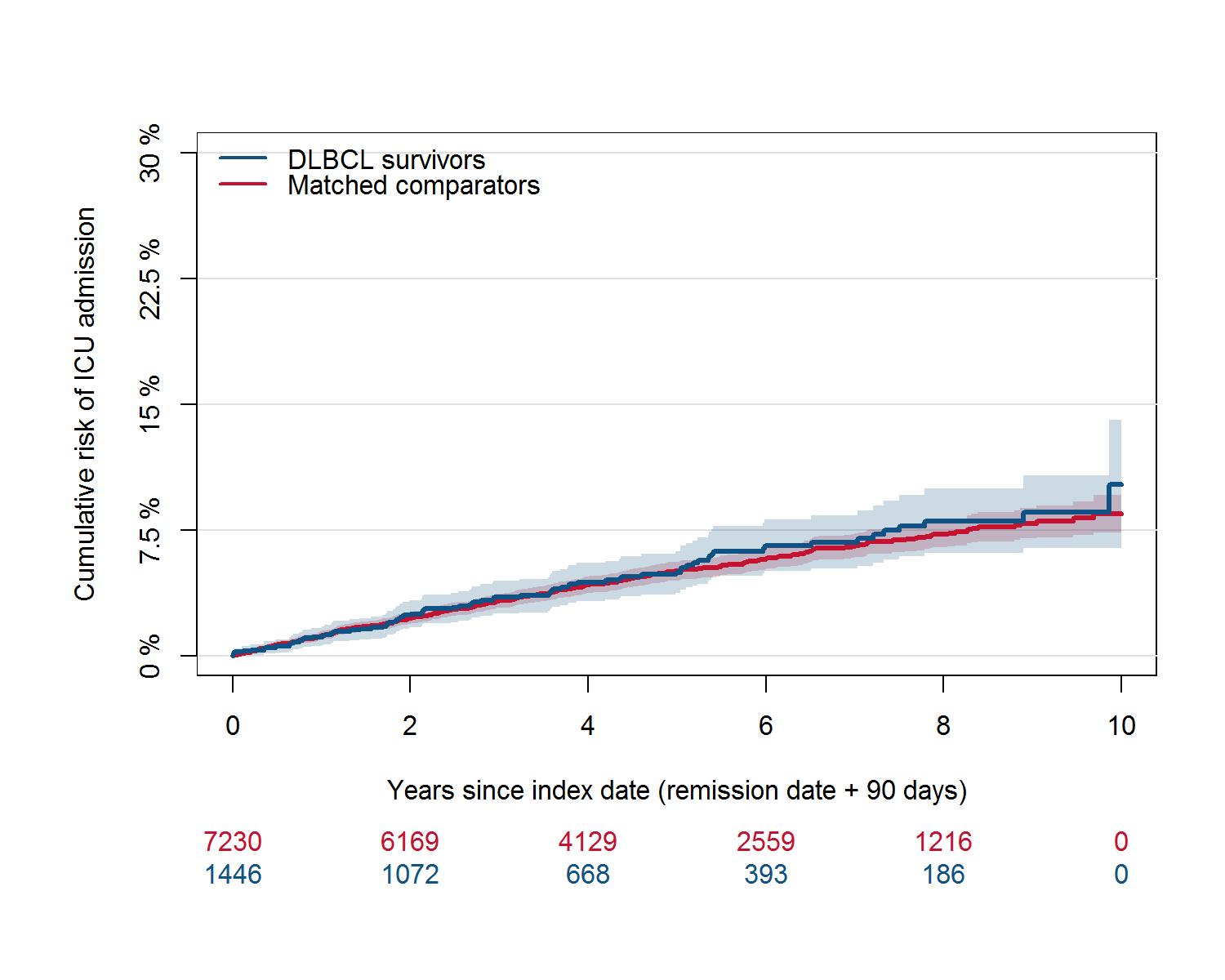


Tables

Table S1: ICD-10 chapters and corresponding codes considered in the present study^*^

| Description (ICD-10 chapter) | ICD-10 codes | Abbreviations |
| --- | --- | --- |
| Malignant neoplasms | C00-C96 | Malignant neoplasms |
| Benign neoplams | D00-D48 | Benign neoplams |
| Diseases of the circulatory system | I00-I99 | Circulatory disorders |
| Infectious diseases | A00-B99 | Infections |
| Mental and behavioral disorders | F00-F99 | Mental |
| Diseases of the respiratory system | J00-J99 | Respiratory |
| Diseases of the genitourinary system | N00-N99 | Genitourinary |
| Symptoms, signs and abnormal clinical and laboratory findings, not elsewhere classified | R00-R99 | Unclassified symptoms and signs |
| Injury, poisoning and certain other consequences of external causes | S00-T98 | Injuries |
| Diseases of the blood and blood-forming organs | D50-D89 | Blood |
| Endocrine, nutritional and metabolic diseases | E00-E90 | Endocrine |
| Diseases of the nervous system | G00-G99 | Neurological |
| Diseases of the eye and adnexa or diseases of the ear and mastoid process | H00-H59 and H60-H95 | Eye and Ear |
| Diseases of the digestive system | K00-K93 | Digestive |
| Diseases of the skin and subcutaneous tissue | L00-L99 | Skin |
| Diseases of the musculoskeletal system and connective tissue | M00-M99 | Musculoskeletal |
| Lymphoma | C81-C86, C88, C96.7, C96.9 | Excluded from outpatient visits |

^*^ Chapters not considered complications to DLBCL were excluded: pregnancy, childbirth and the puerperium (O00-O99), certain conditions originating in the perinatal period (P00-P96), congenital malformations, deformations and chromosomal abnormalities (Q00-Q99), external reasons for disease or death (V01-Y98), factors influencing health status and contact with health services (Z00-Z99) and codes for special purposes (U00-U99).

Table S2: Incidence rates (IR) per 100 person-years of inpatient bed days for each considered ICD-10 diagnostic chapter. The incidence rates are computed for both DLBCL survivors and matched comparators and differences between the two groups are assessed by incidence rate ratios (IRR). For the IRR, 95% confidence interval is provided together with a P-value for a test of the IRR being different from one.

|  | **IR - DLBCL** | **IR - comparators** | **IRR** | **pval** |
| --- | --- | --- | --- | --- |
| Malignant neoplasms | 45.7 | 16.7 | 2.73 (2.61-2.85) | P<0.001 |
| Circulatory disorders | 37.5 | 29.6 | 1.27 (1.21-1.32) | P<0.001 |
| Respiratory | 36.9 | 22.2 | 1.67 (1.59-1.74) | P<0.001 |
| Injury | 28.3 | 16.6 | 1.71 (1.62-1.80) | P<0.001 |
| Digestive | 19.8 | 14.0 | 1.41 (1.33-1.50) | P<0.001 |
| Unclassified symptoms and signs | 18.8 | 13.6 | 1.39 (1.30-1.48) | P<0.001 |
| Infections | 13.5 | 9.1 | 1.49 (1.38-1.61) | P<0.001 |
| Mental | 11.8 | 18.5 | 0.64 (0.59-0.69) | P<0.001 |
| Musculoskeletal | 11.0 | 9.9 | 1.11 (1.02-1.21) | 0.011 |
| Genitourinary | 10.3 | 9.7 | 1.06 (0.97-1.15) | 0.168 |
| Endocrine | 5.9 | 6.3 | 0.94 (0.84-1.05) | 0.300 |
| Skin | 4.2 | 2.0 | 2.16 (1.87-2.48) | P<0.001 |
| Neurological | 3.9 | 7.1 | 0.54 (0.47-0.62) | P<0.001 |
| Benign neoplams | 3.6 | 2.4 | 1.46 (1.26-1.69) | P<0.001 |
| Blood | 2.3 | 2.3 | 1.01 (0.84-1.20) | 0.930 |
| Eye and ear | 1.0 | 0.8 | 1.23 (0.93-1.60) | 0.143 |

Table S3: The five most frequent diagnoses of each ICD-10 chapter associated with inpatient hospital visits among DLBCL survivors. For each diagnosis, the difference between DLBCL survivors and matched comparators was assessed through incidence rate ratios (IRRs) and corresponding 95% confidence interval. A ”*” sign indicate that the IRR is significantly different from 1. Only ICD-10 chapters differing significantly between DLBCL survivors and comparators were considered. Only diagnoses that were observed in at least five survivors are listed. Rates are reported in bed days per 100 person-years.

| ICD chapter | Diagnosis | DLBCL rate | IRR |
| --- | --- | --- | --- |
| Infections | Other sepsis (A41) | 6.3 | 1.44 (1.29-1.61)* |
|  | Bacterial infection of unspecified site (A49) | 1.9 | 1.18 (0.97-1.44) |
|  | Erysipelas (A46) | 1.4 | 1.17 (0.93-1.47) |
|  | Zoster [herpes zoster] (B02) | 0.6 | 4.00 (2.65-6.00)* |
|  | Other gastroenteritis and colitis of infectious and unspecified origin (A09) | 0.6 | 1.62 (1.11-2.30)* |
| Malignant neoplasms | Non-follicular lymphoma (C83) | 22.7 | 92.16 (74.95-114.83)* |
|  | Malignant neoplasm of bronchus and lung (C34) | 4.0 | 1.90 (1.64-2.18)* |
|  | Secondary malignant neoplasm of respiratory and digestive organs (C78) | 1.3 | 1.67 (1.30-2.12)* |
|  | Secondary malignant neoplasm of other and unspecified sites (C79) | 1.3 | 1.78 (1.38-2.27)* |
|  | Lymphoid leukaemia (C91) | 1.3 | 29.63 (17.84-52.52)* |
| Benign neoplams | Neoplasm of uncertain or unknown behaviour of brain and central nervous system (D43) | 0.8 | 6.50 (4.35-9.76)* |
| Circulatory disorders | Heart failure (I50) | 9.6 | 3.03 (2.74-3.34)* |
|  | Atrial fibrillation and flutter (I48) | 5.7 | 1.27 (1.13-1.43)* |
|  | Cerebral infarction (I63) | 3.8 | 1.28 (1.11-1.47)* |
|  | Nonrheumatic aortic valve disorders (I35) | 3.3 | 2.90 (2.45-3.42)* |
|  | Atherosclerosis (I70) | 2.5 | 2.25 (1.86-2.69)* |
| Respiratory | Pneumonia, organism unspecified (J18) | 15.6 | 2.27 (2.11-2.45)* |
|  | Respiratory failure, not elsewhere classified (J96) | 6.7 | 1.51 (1.35-1.67)* |
|  | Bacterial pneumonia, not elsewhere classified (J15) | 6.0 | 1.81 (1.61-2.03)* |
|  | Other chronic obstructive pulmonary disease (J44) | 2.8 | 0.69 (0.59-0.81)* |
|  | Pleural effusion, not elsewhere classified (J90) | 0.8 | 1.78 (1.29-2.41)* |
| Digestive | Paralytic ileus and intestinal obstruction without hernia (K56) | 3.7 | 2.12 (1.82-2.46)* |
|  | Diverticular disease of intestine (K57) | 1.7 | 3.64 (2.84-4.63)* |
|  | Other functional intestinal disorders (K59) | 1.1 | 1.42 (1.09-1.84)* |
|  | Cholelithiasis (K80) | 1.1 | 1.04 (0.80-1.35) |
|  | Ventral hernia (K43) | 1.1 | 3.64 (2.67-4.94)* |
| Skin | Cutaneous abscess, furuncle and carbuncle (L02) | 1.0 | 3.24 (2.36-4.40)* |
| Musculoskeletal | Coxarthrosis [arthrosis of hip] (M16) | 2.4 | 1.37 (1.14-1.63)* |
|  | Gonarthrosis [arthrosis of knee] (M17) | 1.7 | 1.14 (0.92-1.40) |
|  | Dorsalgia (M54) | 0.9 | 1.58 (1.17-2.11)* |
|  | Other spondylopathies (M48) | 0.8 | 0.68 (0.50-0.90)* |
|  | Other soft tissue disorders, not elsewhere classified (M79) | 0.8 | 2.43 (1.73-3.37)* |
| Unclassified symptoms and signs | Fever of other and unknown origin (R50) | 4.2 | 4.88 (4.14-5.75)* |
|  | Abdominal and pelvic pain (R10) | 3.3 | 1.94 (1.65-2.27)* |
|  | Abnormalities of breathing (R06) | 2.1 | 1.51 (1.24-1.82)* |
|  | Shock, not elsewhere classified (R57) | 1.3 | 8.17 (5.85-11.50)* |
|  | Pain, not elsewhere classified (R52) | 1.0 | 1.32 (1.00-1.72)* |
| Injury | Fracture of forearm (S52) | 6.4 | 11.88 (10.04-14.11)* |
|  | Complications of internal orthopaedic prosthetic devices, implants and grafts (T84) | 5.7 | 2.53 (2.23-2.87)* |
|  | Fracture of femur (S72) | 4.5 | 1.39 (1.22-1.58)* |
|  | Other complications of surgical and medical care, not elsewhere classified (T88) | 2.5 | 2.23 (1.85-2.67)* |
|  | Fracture of lower leg, including ankle (S82) | 2.1 | 2.14 (1.75-2.60)* |

Table S4: Incidence rate ratios (DLBCL survivors vs. matched comparators) and corresponding 95% confidence intervals for inpatient bed days stratified by ICD-10 chapter. The incidence rate ratio is computed in three different time periods: 0-2 years, 2-5 years, and 5-10 years after response evaluation.

| ICD-10 chapter | IRR (0-2 yrs) | IRR (2-5 yrs) | IRR (5-10 yrs) |
| --- | --- | --- | --- |
| Skin | 5.7(4.6-7.1) | 0.9(0.6-1.2) | 2.0(1.4-2.8) |
| Malignant neoplasms | 4.2(4.0-4.5) | 1.2(1.0-1.3) | 1.5(1.3-1.7) |
| Eye and ear | 3.4(2.3-4.9) | 1.2(0.6-2.0) | 0.3(0.1-0.7) |
| Injury | 2.5(2.3-2.7) | 1.2(1.1-1.3) | 1.4(1.3-1.6) |
| Respiratory | 2.0(1.8-2.1) | 1.6(1.4-1.7) | 1.6(1.4-1.8) |
| Unclassified symptoms and signs | 1.8(1.6-2.0) | 1.0(0.9-1.1) | 1.2(1.0-1.3) |
| Digestive | 1.7(1.5-1.9) | 0.9(0.8-1.0) | 1.4(1.2-1.6) |
| Infections | 1.6(1.4-1.8) | 1.3(1.1-1.5) | 1.3(1.1-1.5) |
| Circulatory disorders | 1.2(1.1-1.3) | 1.6(1.5-1.7) | 1.1(0.9-1.2) |
| Genitourinary | 1.2(1.0-1.3) | 1.5(1.3-1.7) | 0.7(0.5-0.8) |
| Endocrine | 1.1(0.9-1.2) | 0.6(0.5-0.8) | 1.1(0.8-1.4) |
| Blood | 1.0(0.8-1.3) | 1.6(1.2-2.2) | 0.7(0.4-1.0) |
| Benign neoplams | 1.0(0.7-1.2) | 2.3(1.8-2.9) | 4.4(3.1-6.3) |
| Neurological | 0.9(0.8-1.1) | 0.3(0.2-0.3) | 0.3(0.2-0.4) |
| Musculoskeletal | 0.9(0.8-1.1) | 1.0(0.9-1.1) | 0.9(0.8-1.1) |
| Mental | 0.7(0.6-0.8) | 0.6(0.5-0.6) | 0.3(0.2-0.4) |

Table S5: Incidence rates (IR) per 100 person-years of outpatient visits for each considered ICD-10 diagnostic chapter. The incidence rates are computed for both DLBCL survivors and matched comparators and differences between the two groups are assessed by incidence rate ratios (IRR). For the IRR, 95% confidence interval is provided together with a P-value for a test of the IRR being different from one

|  | **IR - DLBCL** | **IR - comparators** | **IRR** | **P** |
| --- | --- | --- | --- | --- |
| Musculoskeletal | 14.6 | 12.5 | 1.17 (1.09-1.26) | P<0.001 |
| Unclassified symptoms and signs | 11.9 | 8.4 | 1.41 (1.30-1.53) | P<0.001 |
| Circulatory disorders | 11.9 | 9.4 | 1.27 (1.17-1.37) | P<0.001 |
| Eye and ear | 10.5 | 8.4 | 1.25 (1.15-1.36) | P<0.001 |
| Injury | 9.4 | 8.5 | 1.10 (1.01-1.20) | 0.029 |
| Malignant neoplasms | 8.7 | 6.6 | 1.31 (1.19-1.44) | P<0.001 |
| Digestive | 7.7 | 5.9 | 1.30 (1.17-1.43) | P<0.001 |
| Genitourinary | 6.1 | 4.5 | 1.35 (1.21-1.51) | P<0.001 |
| Endocrine | 4.6 | 3.3 | 1.39 (1.22-1.58) | P<0.001 |
| Benign neoplams | 3.9 | 2.8 | 1.37 (1.19-1.58) | P<0.001 |
| Respiratory | 3.7 | 2.8 | 1.32 (1.14-1.51) | P<0.001 |
| Neurological | 3.5 | 3.6 | 0.98 (0.85-1.13) | 0.773 |
| Skin | 2.6 | 1.6 | 1.62 (1.36-1.93) | P<0.001 |
| Mental | 2.1 | 2.2 | 0.94 (0.78-1.13) | 0.509 |
| Infections | 2.1 | 0.9 | 2.26 (1.84-2.77) | P<0.001 |
| Blood | 1.6 | 0.8 | 2.05 (1.62-2.57) | P<0.001 |

Table S6: The five most frequent diagnoses of each ICD-10 chapter associated with outpatient visits among DLBCL survivors. For each diagnosis, the difference between DLBCL survivors and matched comparators was assessed through incidence rate ratios (IRRs) and corresponding 95% confidence interval. A ”*” sign indicate that the IRR is significantly different from 1. Only ICD-10 chapters differing significantly between DLBCL survivors and comparators were considered. Only diagnoses that were observed in at least five survivors are listed. Rates are reported in visits per 100 person-years.

| ICD chapter | Diagnosis | DLBCL rate | IRR |
| --- | --- | --- | --- |
| Infections | Human immunodeficiency virus [HIV] disease resulting in other conditions (B23) | 0.4 | 4.44 (2.52-7.70)* |
|  | Erysipelas (A46) | 0.3 | 1.54 (0.86-2.59) |
|  | Chronic viral hepatitis (B18) | 0.2 | 3.04 (1.51-5.85)* |
|  | Other sepsis (A41) | 0.2 | 1.43 (0.70-2.66) |
| Malignant neoplasms | Other malignant neoplasms of skin (C44) | 1.8 | 2.68 (2.14-3.34)* |
|  | Malignant neoplasm of bronchus and lung (C34) | 1.7 | 2.36 (1.88-2.95)* |
|  | Malignant neoplasm of prostate (C61) | 0.9 | 0.78 (0.59-1.02) |
|  | Malignant neoplasm of breast (C50) | 0.8 | 0.98 (0.72-1.30) |
|  | Lymphoid leukaemia (C91) | 0.3 | 3.25 (1.85-5.56)* |
| Benign neoplams | Benign neoplasm of colon, rectum, anus and anal canal (D12) | 1.0 | 0.95 (0.73-1.23) |
|  | Benign lipomatous neoplasm (D17) | 0.4 | 1.93 (1.21-2.96)* |
|  | Benign neoplasm of brain and other parts of central nervous system (D33) | 0.3 | 8.29 (3.99-17.79)* |
|  | Other benign neoplasms of skin (D23) | 0.2 | 1.79 (0.97-3.11)* |
|  | Benign neoplasm of urinary organs (D30) | 0.2 | 1.58 (0.84-2.76) |
| Blood | Other anaemias (D64) | 0.5 | 1.40 (0.93-2.03) |
|  | Sarcoidosis (D86) | 0.5 | 7.32 (4.29-12.62)* |
|  | Iron deficiency anaemia (D50) | 0.3 | 2.23 (1.22-3.88)* |
| Endocrine | Type 2 diabetes mellitus (E11) | 1.8 | 1.45 (1.17-1.78)* |
|  | Other nontoxic goitre (E04) | 0.4 | 1.51 (0.95-2.30) |
|  | Type 1 diabetes mellitus (E10) | 0.3 | 1.03 (0.61-1.66) |
|  | Postprocedural endocrine and metabolic disorders, not elsewhere classified (E89) | 0.3 | 6.20 (3.18-12.12)* |
|  | Thyrotoxicosis [hyperthyroidism] (E05) | 0.2 | 1.08 (0.60-1.82) |
| Eye and ear | Senile cataract (H25) | 2.7 | 1.14 (0.96-1.34) |
|  | Other hearing loss (H91) | 2.4 | 1.26 (1.06-1.50)* |
|  | Other retinal disorders (H35) | 0.9 | 1.26 (0.93-1.67) |
|  | Conductive and sensorineural hearing loss (H90) | 0.7 | 1.97 (1.39-2.73)* |
|  | Other cataract (H26) | 0.5 | 1.49 (1.00-2.14)* |
| Circulatory disorders | Atrial fibrillation and flutter (I48) | 2.3 | 1.24 (1.04-1.48)* |
|  | Heart failure (I50) | 1.9 | 2.94 (2.35-3.66)* |
|  | Nonrheumatic aortic valve disorders (I35) | 1.0 | 1.54 (1.16-2.02)* |
|  | Aortic aneurysm and dissection (I71) | 0.7 | 1.73 (1.23-2.39)* |
|  | Other peripheral vascular diseases (I73) | 0.7 | 1.44 (1.02-1.99)* |
| Respiratory | Other chronic obstructive pulmonary disease (J44) | 1.2 | 1.11 (0.86-1.42) |
|  | Pneumonia, organism unspecified (J18) | 0.7 | 2.18 (1.51-3.10)* |
|  | Respiratory failure, not elsewhere classified (J96) | 0.3 | 1.27 (0.71-2.11) |
|  | Asthma (J45) | 0.3 | 0.74 (0.43-1.21) |
|  | Diseases of vocal cords and larynx, not elsewhere classified (J38) | 0.2 | 1.89 (0.92-3.62) |
| Digestive | Inguinal hernia (K40) | 0.9 | 0.98 (0.73-1.29) |
|  | Other functional intestinal disorders (K59) | 0.6 | 1.78 (1.22-2.53)* |
|  | Ventral hernia (K43) | 0.4 | 3.44 (2.11-5.48)* |
|  | Diverticular disease of intestine (K57) | 0.4 | 0.88 (0.56-1.31) |
|  | Other noninfective gastroenteritis and colitis (K52) | 0.4 | 1.69 (1.05-2.62)* |
| Skin | Ulcer of lower limb, not elsewhere classified (L97) | 0.3 | 1.37 (0.83-2.15) |
|  | Other disorders of skin and subcutaneous tissue, not elsewhere classified (L98) | 0.3 | 1.99 (1.10-3.44)* |
|  | Other dermatitis (L30) | 0.3 | 3.12 (1.66-5.65)* |
|  | Psoriasis (L40) | 0.2 | 3.51 (1.81-6.58)* |
|  | Skin changes due to chronic exposure to nonionizing radiation (L57) | 0.2 | 1.25 (0.59-2.36) |
| Musculoskeletal | Coxarthrosis [arthrosis of hip] (M16) | 1.4 | 1.34 (1.05-1.69)* |
|  | Osteoporosis without pathological fracture (M81) | 1.2 | 1.29 (1.00-1.65)* |
|  | Gonarthrosis [arthrosis of knee] (M17) | 1.1 | 0.88 (0.68-1.13) |
|  | Seropositive rheumatoid arthritis (M05) | 1.0 | 3.16 (2.30-4.31)* |
|  | Other disorders of bone density and structure (M85) | 0.9 | 2.62 (1.91-3.54)* |
| Genitourinary | Chronic kidney disease (N18) | 0.7 | 0.93 (0.66-1.27) |
|  | Calculus of kidney and ureter (N20) | 0.6 | 1.62 (1.10-2.32)* |
|  | Other disorders of urinary system (N39) | 0.6 | 1.52 (1.03-2.17)* |
|  | Obstructive and reflux uropathy (N13) | 0.5 | 3.86 (2.47-5.97)* |
|  | Cystitis (N30) | 0.4 | 1.17 (0.76-1.73) |
| Unclassified symptoms and signs | Abdominal and pelvic pain (R10) | 1.5 | 1.76 (1.39-2.20)* |
|  | Abnormalities of breathing (R06) | 1.2 | 1.98 (1.52-2.55)* |
|  | Abnormal findings on diagnostic imaging of lung (R91) | 1.0 | 2.04 (1.52-2.70)* |
|  | Pain, not elsewhere classified (R52) | 0.9 | 1.88 (1.39-2.50)* |
|  | Other symptoms and signs involving the urinary system (R39) | 0.7 | 0.94 (0.68-1.27) |
| Injury | Fracture of forearm (S52) | 0.8 | 1.14 (0.83-1.53) |
|  | Complications of internal orthopaedic prosthetic devices, implants and grafts (T84) | 0.8 | 1.75 (1.25-2.41)* |
|  | Fracture of lower leg, including ankle (S82) | 0.7 | 1.79 (1.25-2.49)* |
|  | Fracture of shoulder and upper arm (S42) | 0.6 | 1.31 (0.90-1.85) |
|  | Fracture of femur (S72) | 0.5 | 1.95 (1.28-2.89)* |

Table S7: Incidence rate ratios (DLBCL survivors vs. matched comparators) and corresponding 95% confidence intervals for outpatient visits stratified by ICD-10 chapter. The incidence rate ratio is computed in three different time periods: 0-2 years, 2-5 years, and 5-10 years after response evaluation.

| ICD-10 chapter | IRR (0-2 yrs) | IRR (2-5 yrs) | IRR (5-10 yrs) |
| --- | --- | --- | --- |
| Infections | 3.4(2.5-4.5) | 2.1(1.5-3.0) | 2.7(1.8-4.0) |
| Blood | 2.5(1.8-3.4) | 2.5(1.7-3.5) | 1.3(0.8-2.2) |
| Skin | 2.0(1.6-2.6) | 1.6(1.2-2.1) | 1.1(0.7-1.6) |
| Benign neoplams | 1.8(1.4-2.1) | 1.4(1.1-1.7) | 1.4(1.0-1.8) |
| Endocrine | 1.7(1.4-2.0) | 1.2(1.0-1.5) | 1.2(0.9-1.5) |
| Genitourinary | 1.7(1.4-2.0) | 1.2(1.0-1.4) | 1.0(0.8-1.3) |
| Digestive | 1.6(1.4-1.9) | 1.0(0.8-1.2) | 1.2(1.0-1.5) |
| Unclassified symptoms and signs | 1.6(1.4-1.8) | 1.3(1.1-1.4) | 1.3(1.1-1.5) |
| Respiratory | 1.4(1.2-1.8) | 1.2(1.0-1.5) | 1.0(0.8-1.4) |
| Circulatory disorders | 1.3(1.2-1.5) | 1.3(1.2-1.5) | 1.3(1.1-1.6) |
| Musculoskeletal | 1.3(1.2-1.4) | 1.1(1.0-1.2) | 1.2(1.1-1.4) |
| Malignant neoplasms | 1.3(1.1-1.5) | 1.1(0.9-1.3) | 1.2(1.0-1.4) |
| Eye and ear | 1.3(1.1-1.4) | 1.3(1.1-1.5) | 1.1(0.9-1.3) |
| Injury | 1.2(1.1-1.4) | 1.0(0.9-1.2) | 1.1(1.0-1.3) |
| Neurological | 1.2(1.0-1.4) | 0.9(0.7-1.1) | 0.8(0.6-1.1) |
| Mental | 0.9(0.6-1.2) | 0.8(0.6-1.1) | 1.1(0.8-1.5) |
